# Einfluss der COVID-19-Pandemie auf die psychische Gesundheit während der Peripartalzeit – eine narrative Übersicht

**DOI:** 10.1007/s00278-023-00646-w

**Published:** 2023-02-23

**Authors:** S. Gries, N. S. Teichmann, F. M. L. Beck-Hiestermann, B. Strauß, A. Gumz

**Affiliations:** 1 Psychologische Hochschule Berlin, Am Köllnischen Park 2, 10179 Berlin, Deutschland; 2 Universitätsklinikum Jena, Institut für Psychosoziale Medizin, Psychotherapie und Psychoonkologie (IPMPP), Jena, Deutschland

**Keywords:** COVID-19, Schwangerschaft, Pandemie, Peripartalzeit, Postpartale Depression, COVID-19, Pregnancy, Pandemic, Peripartum period, Postpartum depression

## Abstract

**Hintergrund:**

Die Auswirkungen der COVID-19-Pandemie und die darauffolgenden Maßnahmen der Regierung waren mit drastischen Einschnitten in das Leben nahezu aller Menschen assoziiert. Betroffen waren v. a. vulnerable Gruppen, darunter schwangere Frauen und Mütter in der postpartalen Phase.

**Fragestellung:**

Ziel des Reviews war die Untersuchung des Einflusses der COVID-19-Pandemie auf die psychische Gesundheit von schwangeren Frauen und Mütter in der Postpartalzeit anhand von Symptomen bezüglich Stress, Angst, Depression und posttraumatischer Belastungsstörung (PTBS). Weiterhin wurde nach Risiko- und Schutzfaktoren gesucht, die zur Entstehung oder zur Vorbeugung postpartaler psychischer Erkrankungen während der Pandemie beitragen.

**Material und Methode:**

Die Literatursuche erfolgte via PsycArticles, PsycINFO, PSYNDEX und Ovid Medline von April bis Juni 2021. Extrahiert wurden Angst‑, Depressions‑, Stress- und PTBS-Symptome sowie potenzielle Risiko- und Schutzfaktoren.

**Ergebnisse:**

Es wurden 19 relevante Studien mit 44.709 Teilnehmerinnen aufgenommen. Beinahe alle Studien verzeichneten einen Anstieg der Angst‑, Depressions‑, Stress- und PTBS-Symptome während der Pandemie. Finanzielle, intrafamiliäre Stressoren sowie die Sorge um das Kind wurden als Risikofaktor für die Entstehung postpartaler psychischer Erkrankungen während der Pandemie identifiziert. Die Zufriedenheit mit der Paarbeziehung schützte augenscheinlich vor Stress- und Depressionssymptomen. Angstsymptome wurden u. a. durch ein erhöhtes Ausmaß physischer Aktivität und die wahrgenommene soziale Unterstützung reduziert.

**Schlussfolgerungen:**

Zukünftigen Untersuchungen wird empfohlen, die Risikofaktoren für die Entwicklung postpartaler psychischer Erkrankungen noch genauer zu untersuchen. Zudem sollten Präventionsprogramme für das medizinische Personal sowie Nachsorge- und Therapieprogramme für betroffene Mütter entwickelt werden, um schwere Verläufe zu verhindern.

## Abstract (en)

**Background:**

The impact of the coronavirus disease 2019 (COVID-19) pandemic and the subsequent measures by the government were associated with a drastic disruption of the lives of nearly everyone. Vulnerable groups were particularly affected, including pregnant women and mothers in the postpartum period.

**Objective:**

The aim of the review was to examine the impact of the COVID-19 pandemic on the mental health of pregnant women and mothers in the postpartum period based on stress, anxiety, depression and posttraumatic stress disorder (PTSD) symptoms. Furthermore, risk and protective factors that contributed to the development or prevention of postpartum mental illnesses during the pandemic were identified.

**Methods:**

A literature search was conducted via PsycArticles, PsycINFO, PSYNDEX, and Ovid Medline from April to June 2021. Data on anxiety, depression, stress, and PTSD symptoms, as well as potential risk and protective factors were extracted.

**Results:**

A total of 19 relevant studies with 44,709 participants were included. Nearly all studies recorded an increase in anxiety, depression, stress, and PTSD symptoms during the pandemic. Financial, intrafamilial stressors and concern for the child were identified as risk factors for the development of postpartum mental illnesses during the pandemic. Satisfaction with the couple relationship was apparently protective against stress and depression symptoms. Anxiety symptoms were reduced, for example, by increased levels of physical activity as well as perceived social support.

**Conclusion:**

Future studies are recommended to further investigate the risk factors for the development of postpartum mental illnesses. In addition, prevention programs for medical personnel as well as aftercare and therapy programs for affected mothers should be developed in order to prevent severe progression.

Die COVID-19-Pandemie führt weltweit zu gesundheitlichen und ökonomischen Problemen in der Gesellschaft (World Health Organization 2020). Die damit verbundenen Einschränkungen im Gesundheitssystem erhöhen das Ausmaß von wahrgenommenem Stress und die Auftretenshäufigkeit psychischer Erkrankungen (Cooke et al. 2020; Shi et al. 2020) und gefährden v. a. Personen mit erhöhter Vulnerabilität. Dazu zählen auch potenziell schwangere Frauen und Frauen in der postpartalen Phase (Dubber et al. 2015). Psychische Erkrankungen bei Schwangeren können sowohl das Wohlbefinden der werdenden Mutter als auch die Gesundheit des Kindes negativ beeinflussen (Kramer et al. 2009; Yu et al. 2013).

## Psychische Erkrankungen während der Schwangerschaft und Postpartalzeit

Eine Schwangerschaft und die Zeit vor der Geburt (präpartal), der Zeitraum um die Geburt herum (peripartal) und die Phase nach der Geburt (postpartal) sind für viele Frauen ein emotional bedeutsamer Lebensabschnitt, der mit psychologischen und physiologischen Veränderungen einhergeht. Bisherige Studien weisen darauf hin, dass psychische Erkrankungen der Mutter das Risiko einer Frühgeburt oder Schwangerschaftsvergiftung (Präeklampsie) erhöhen (Kramer et al. 2009; Orr et al. 2007). Weiterhin können postpartale psychische Erkrankungen die Mutter-Kind-Bindung nach der Geburt beeinflussen (Cierpka 2014; Dubber et al. 2015; Moehler et al. 2006).

### Merke.

Postpartale psychische Erkrankungen beeinflussen Mutter-Kind-Bindung.

### Stress

Als peripartaler Stress gelten sowohl psychischer und emotionaler Stress als auch belastende Lebensereignisse (Mohler et al. 2006). Bereits während der Schwangerschaft kann sich vermehrt wahrgenommener Stress negativ auf die Entwicklung des Fetus auswirken. Dazu gehört der Einfluss von Stress auf das Wachstum der kognitiven und emotionalen Strukturen im Gehirn sowie auf das Geburtsgewicht (La Marca-Ghaemmaghami und Ehlert 2015; Tarabulsy et al. 2014; Van den Bergh et al. 2020). Weiterhin korrelierte mütterlicher emotionaler Stress während der Schwangerschaft positiv mit späteren Stressreaktionen des Kindes (Zietlow et al. 2019). Die Prävalenzraten für wahrgenommenen Stress während der Schwangerschaft variieren stark zwischen 5,5 und 78 % (Engidaw et al. 2019). Die Auswirkungen der COVID-19-Pandemie beeinflussen besonders die vulnerablen Gruppen der Gesellschaft und führen bei schwangeren Frauen zu außergewöhnlichem Stresserleben (Matvienko-Sikar et al. 2021). Einige Forscher unterteilen den COVID-19-bezogenen Stress in 2 Kategorien: a) „preparedness stress“, der das Gefühl beschreibt, aufgrund der Pandemie nicht ausreichend auf die Geburt vorbereitet zu sein, und b) „perinatal infection stress“, d. h. Angst vor einer peripartalen COVID-19-Infektion (Ilska et al. 2021; Schaal et al. 2021). Aus einer italienischen Studie geht hervor, dass bei schwangeren Frauen Preparedness stress eher erhöhte Werte generalisierter Angst und der Perinatal infection stress eher erhöhte Depressionswerte vorhersagt (Penengo et al. 2021).

### Angst und Depressionen

Die Prävalenzraten prä- und postpartaler Angststörungen schwanken in der Literatur zwischen 10 und 25 % (Dennis et al. 2017; Fawcett et al. 2019; Howard und Khalifeh 2020). Einige Frauen entwickeln während der Schwangerschaft eine starke Angst vor der Geburt, häufig in Kombination mit Panikattacken und Schlafstörungen (Fenwick et al. 2009; O’Connell et al. 2017). Dies hat sowohl einen negativen Einfluss auf die Bewältigung des Alltags als auch auf die Verarbeitung der Wehen und der Geburt. Darüber hinaus stehen präpartale Ängste im Zusammenhang mit höheren Raten von Essstörungen und einem erhöhten Suizid- sowie Frühgeburtenrisiko (George et al. 2013; Micali et al. 2011; Sanchez et al. 2013). Zudem ist die präpartale Angst ein signifikanter Risikofaktor für das Entstehen einer postpartalen Depression (Faisal-Cury und Menezes 2012). Auf der Seite des ungeborenen Kindes erhöhen präpartale Ängste das Risiko für eine schlechtere kognitive Entwicklung sowie eine gestörte Mutter-Kind-Bindung (Catov et al. 2010; Dubber et al. 2015; Keim et al. 2011). Eine italienische Studie legt nahe, dass die COVID-19-Pandemie das psychische Wohlbefinden italienischer Schwangerer v. a. im Hinblick auf Angsterkrankungen deutlich verschlechtert hat (Colli et al. 2021).

Prä-, peri- und postpartale Depressionen gleichen symptomatisch einer depressiven Episode und betreffen laut Studienlage zwischen 10 und 15 % der schwangeren Frauen (Bennett et al. 2004; Riecher-Rössler 1997; Yim et al. 2015). Dabei galten Depressionen in der Vergangenheit, niedriges Einkommen, mangelnde soziale Unterstützung, häusliche Gewalt sowie belastende Lebensereignisse als signifikante Risikofaktoren. Frauen in der Spätschwangerschaft und Frauen, die im letzten Trimester Angstsymptome aufwiesen, hatten ebenfalls ein erhöhtes Risiko, depressive Symptome zu entwickeln (Hübner-Liebermann et al. 2012; Pampaka et al. 2018; Sunnqvist et al. 2019; Zaers et al. 2008). Präpartale Depressionen korrelierten signifikant positiv mit einem erhöhten Frühgeburtsrisiko und geringerem Geburtsgewicht des Kindes (Hübner-Liebermann et al. 2012; Jarde et al. 2016). Zudem gilt eine präpartale Depression als ein Prädiktor für postpartale Depression („postPD“; Faisal-Cury und Menezes 2012). Die Symptome einer „postPD“ gleichen denen einer depressiven Episode gemäß der ICD-10, wobei Versagensängste, Insuffizienz‑, Scham- und Schuldgefühle in Bezug auf das Muttersein hinzukommen können (Dorsch und Rohde 2016; Hübner-Liebermann et al. 2012). Differenzialdiagnostisch abzugrenzen ist diese von dem häufig auftretenden „Baby Blues“ (passagere postpartale Dysphorie), ein Zustand, der sich u. a. durch Niedergeschlagenheit und Stimmungslabilität ausdrückt und innerhalb der Tage 1 bis 10 postpartum überwunden sein sollte (Lasch und Fillenberg 2017; Riecher-Rössler 1997). Als Risikofaktoren für die Entwicklung einer „postPD“ (Beginn meist ab dem 14. Tag postpartum) gelten eine geringe soziale Unterstützung, eine konfliktreiche Partnerschaft, häusliche Gewalt, Missbrauchserfahrung, frühere affektive Störungen oder Angststörungen sowie chronische Erkrankungen (Bloch et al. 2005; O’Hara und McCabe 2013; Werner et al. 2015). Die „postPD“ kann, mediiert durch gestörte Bonding-Prozesse zwischen Mutter und Kind, negative Auswirkungen auf die kognitive und psychoaffektive Entwicklung des Kindes haben (Cogill et al. 1986; Roux et al. 2002).

### Posttraumatische Belastungsstörungen

Eine posttraumatische Belastungsreaktion kann als Folgereaktion eines traumatischen Ereignisses verstanden werden, wobei Letzteres eine Situation mit außergewöhnlicher Bedrohung oder katastrophenartigem Ausmaß beschreibt (Dorsch et al. 2017). Bis zu 33 % der Frauen erleben die Entbindung als traumatisches Ereignis (Maggioni et al. 2006; Söderquist et al. 2009); eine posttraumatische Belastungsstörung (PTBS) entwickelt sich mit einer Prävalenz von 2–7 % (Söderquist et al. 2009). Symptomatisch äußert sich eine PTBS im Wiedererleben des Traumas mit Intrusionen und Flashbacks, durch Vermeidungsverhalten, Gereiztheit und das Gefühl innerer Stumpfheit (Dorsch und Rohde 2016). Söderquist et al. (2009) nennen Depressionen während der Frühschwangerschaft und starke Angst vor der Geburt als Risikofaktoren für die Entwicklung einer PTBS. Ebenso stellen traumatische Vorerfahrungen der Frauen einen Risikofaktor dar, wobei die Prävalenz für diese Gruppe 6–8 % beträgt (Seng et al. 2010).

#### Merke.

Bis zu 33 % der schwangeren Frauen erleben die Entbindung als traumatisches Ereignis.

## Review

### Ziele

Die COVID-19-Pandemie stellt eine historisch kaum vergleichbare Situation dar, entsprechend existiert wenig Referenzliteratur. In einem aktuellen Review gaben schwangere Frauen während der derzeitigen Phase der Coronapandemie stark erhöhte Depressions- und Angstwerte an (Campos-Garzón et al. 2021). Ziele des vorliegenden Reviews waren zunächst die Untersuchung des Einflusses der COVID-19-Pandemie auf die psychische Gesundheit von schwangeren Frauen und Frauen in der Postpartalzeit anhand von Stress‑, Angst‑, Depressions- sowie PTBS-Symptomen. Weiterhin wurde nach Risiko- und Schutzfaktoren gesucht, die zur Entstehung oder zur Vorbeugung einer postpartalen psychischen Erkrankung während der Pandemie beitragen.

### Material und Methoden

#### Ein- und Ausschlusskriterien

Eingeschlossen wurden Studien, wenn sie a) mindestens ein Merkmal psychischer Gesundheit der schwangeren Frau untersuchten, b) der Erhebungszeitraum während der Pandemie mit Beginn im März 2020 lag, und es sich c) um Primärstudien handelte. Um ein breites Spektrum an Literatur zu erhalten, wurden keine weiteren Einschränkungen festgelegt.

#### Suchstrategie und Studienauswahl

Die Literatursuche erfolgte über die elektronischen Datenbanken PsycArticles, PsycINFO, PSYNDEX sowie Ovid MEDLINE mit der folgenden Schlagwortkombination: (″COVID-19″, ″pandemic″, ″SARS-CoV-2″, ″pregnancy″, ″birth″, ″postpartum″, ″perinatal″, ″depression″, ″anxiety″, ″PTSD″, ″mental health″). Die Suche wurde zwischen April und Juni 2021 durchgeführt. Neben der Datenbanksuche wurden weitere Studien durch das Schneeballsystem (Greenhalgh und Peacock 2005) identifiziert. Die Erstautorin sichtete zunächst die Titel und Abstracts, und im darauffolgenden Schritt sichteten die Erst- und die Zweitautorin die Volltexte unabhängig voneinander.

### Ergebnisse

#### Beschreibung der Studien

**Figure 1:**
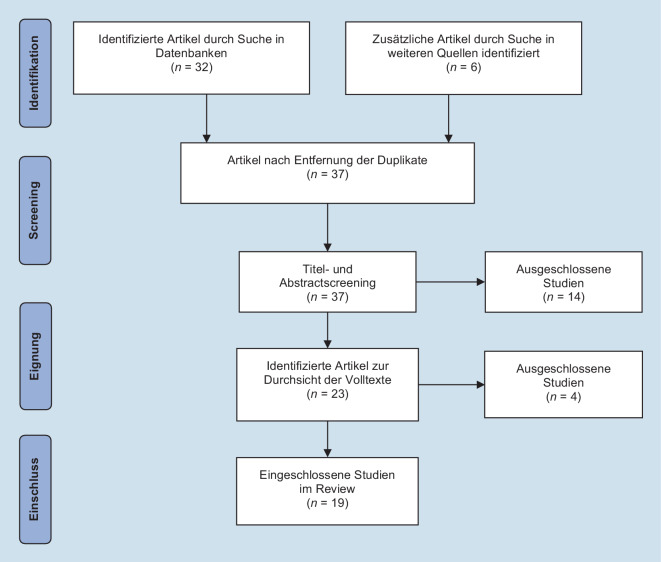

Den Flowchart zu Suchstrategie und Studienauswahl zeigt Abb. 1. Demnach wurden 38 Studien identifiziert und nach Entfernung von Duplikaten 37 Artikel hinsichtlich Titel und Abstract gesichtet. Schlussendlich wurden 19 Volltexte in das Review aufgenommen. Die Eigenschaften der aufgenommenen Studien sind deskriptiv in Tab. 1 zusammengefasst. Die Studien stammen aus den USA (*k* = 4), China (*k* = 3), Italien (*k* = 3), Kanada (*k* = 3), Argentinien (*k* = 1), Äthiopien (*k* = 1), Irland (*k* = 1), Iran (*k* = 1), Israel (*k* = 1) und Frankreich (*k* = 1). Das überwiegende Studiendesign war querschnittlich (*k* = 14). Lediglich 3 Studien wiesen ein längsschnittliches Design auf (Gonzalez-Garcia et al. 2021; López-Morales et al. 2021; Perzow et al. 2021). Die Untersuchung von Pariente et al. (2020) war als Kohortenstudie aufgebaut. Berthelot et al. (2020) kombinierten in ihrer Arbeit ein längsschnittliches Design (erste Kohorte vor der Pandemie) mit einer Fall-Kontroll-Studie (zweite Kohorte während der Pandemie). Die Messzeiträume variierten je nach Studiendesign und Erhebungsland, da die COVID-19-Pandemie in den einzelen Erhebungsländern zu unterschiedlichen Zeitpunkten begonnen hatte. Die Zahl der teilnehmenden Patientinnen betrug insgesamt 44.709 (*n* = 1740 postpartal; *n* = 120 nicht schwanger; *n* = 776 keine Trennung von postpartal und schwanger). In 19 Studien wurden schwangere Frauen rekrutiert, in 5 Studien wurden zusätzlich Frauen befragt, die sich im postpartalen Zeitraum befanden (Basu et al. 2021; Cameron et al. 2020; Gonzalez-Garcia et al. 2021; Molgora und Accordini 2020; Perzow et al. 2021). An einer Studie nahmen ausschließlich Frauen in der postpartalen Phase teil (Pariente et al. 2020), und in einer weiteren Studie wurde der Fokus auf erstgebärende Schwangere gelegt (McMillan et al. 2021). Das Durchschnittsalter der Frauen betrug 31,5 Jahre, wobei die Altersspanne von 18 bis 46 Jahre reichte. In einer Studie wurde kein Mittelwert des Alters angegeben, dort waren 89,1 % der Frauen jünger als 35 Jahre (Liu et al. 2020). In einer weiteren Studie wurde der Median von 30 Jahren für das Alter angegeben (Spannweite von 27 bis 32 Jahre; Wu et al. 2020).

**Table 1:** 

Autoren, Land	Design	Zeitraum	Stichprobe	Variablen Instrumente	Ergebnisse
Basu et al. (2021), USA	Querschnitt	26.05.2020–13.06.2020 (während der Pandemie)	Schwangere (*n* = 5712) und Frauen in der postpartalen Phase (*n* = 1182)	Depression; Angst; PTBS	Höhere Prävalenzen bezüglich Depression, Angst und PTBS im Vergleich zur Allgemeinbevölkerung während COVID-19 und zu Schwangeren/Frauen in der postpartalen Phase vor COVID-19; sign. Korrelation mit erhöhten Depressions‑, Angst- und PTBS-Symptomen: übermäßige Informationssuche, Sorgen in Bezug auf die medizinische Versorgung
				PHQ‑4; IES‑6	
Berthelot et al. (2020), Kanada	Fall-Kontroll-Studie; Längsschnitt	April 2020 (währende der Pandemie) April 2018 bis März 2020 (Präpandemie)	Schwangere Frauen (*n* = 1754)	Depression; Angst; PTBS	COVID-19-Kohorte: höhere Depressions‑, Angst‑, PTBS-Symptome als Prä-COVID-19-Kohorte
				K10; PCL‑5	
Cameron et al. (2020), Kanada	Querschnitt	14.04.2020–28.04.2020 (während der Pandemie)	Schwangere und Frauen in der postpartalen Phase (*n* = 641)	Prä-/postpartale Depression; generalisierte Angst; peripartale Angst	Depressions- und Angstwerte im Vergleich zu Prä-COVID-19 stark erhöht; sign. Korrelationen zwischen Risikofaktoren und Depression bzw. Angst
				EPDS; GAD‑7; PASS; MSPSS	
Dule et al. (2021), Äthiopien	Querschnitt	01.08.2020–15.08.2020 (während der Pandemie)	Schwangere Frauen (*n* = 384)	COVID-19-bezogene Angst	Sign. negative Korrelation zwischen Angst vor COVID-19 und Lebenszufriedenheit
				MSPSS; FCoV-19S	
Effati-Daryani et al. (2020), Iran	Querschnitt	März bis April 2020 (während der Pandemie)	Schwangere Frauen (*n* = 205)	Depression, Stress, Angst	Mit denen vor der Pandemie vergleichbare Prävalenzen in der Pandemie für Depressions‑, Stress- und Angstsymptome
				DASS-21; Fragebogen zu soziodemografischen Merkmalen	
Gonzalez-Garcia et al. (2021), Frankreich	Längsschnitt	06.04.2020–11.05.2020 (während der Pandemie)	Schwangere/Frauen in der postpartalen Phase (während der Schwangerschaft *n* = 90; einen Monat nach der Geburt *n* = 26)	Angst, prä-/postpartale Depression; PTBS	Sign. positive Korrelation zwischen Sorge über die Auswirkungen von COVID-19 auf das Baby und Angst- und Depressionswerten; höhere Angstwerte während der Pandemie im Vergleich zur Kontrollstichprobe vor der Pandemie; keine Unterschiede in den PTBS-Werten
				STAI, EPDS, MSPSS, CBTS	
Lebel et al. (2020), Kanada	Querschnitt	05.04.2020–20.04.2020 (während der Pandemie) 2012–2016 (frühere Kohorten)	Schwangere Frauen (*n* = 1987)	Präpartale Depression, generelle Angst, peripartale Angst	Erhöhte Werte für Angst- und Depressionssymptome im Vergleich zu ähnlicher Kohorte vor der Pandemie und positive Korrelation mit Angst um das Leben der Mutter und des Kindes
				EPDS; Patient-Reported Outcomes Measurement Information System – Anxiety Adult 7‑item short form (PROMIS); PRAQ; Interpersonal Support Evaluation List (ISEL)	
Liu et al. (2020), China	Querschnitt	03.02.2020–09.02.2020 (während der Pandemie)	Schwangere Frauen (*n* = 1947)	Präpartale Angst	Mehr Wissen über COVID-19 und eine rationale Risikowahrnehmung korrelierte negativ mit Angstsymptomen; Symptome einer Infektion korrelierten positiv mit der Wahrscheinlichkeit, Angst zu entwickeln
				SAS	
López-Morales et al. (2021), Argentinien	Längsschnitt	20.03.2020–10.05.2020 (während der Pandemie)	Schwangere (*n* = 102) und nichtschwangere Frauen (*n* = 102)	Depression; Angst	Innerhalb der Quarantänezeit: Anstieg von Angst- und Depressionswerten der Schwangeren stärker als bei Nichtschwangeren
				BDI-II; STAI	
Matvienko-Sikar et al. (2020), Irland	Querschnitt	16.06.2020–17.07.2020 (während der Pandemie) Mai 2019 bis Februar 2020 (vor der Pandemie)	Schwangere Frauen (*n* = 445)	Stress	Kein sign. Anstieg des Stressniveaus
				NuPDQ; MSPSS	
McMillan et al. (2021), USA	Querschnitt	Juni bis Juli 2020 (während der Pandemie)	Erstgebärende schwangere Frauen (*n* = 49)	Depression; Angst; Stress	Verbleiben in Isolation: sign. positive Korrelation mit Depression und Angst; Zunahme von Alkoholkonsum und Gewalt in der Partnerschaft ≙ Prädiktoren für erhöhte Depressions‑, Angst- und Stresswerte
				DASS-21	
Molgora und Accordini (2020), Italien	Querschnitt	01.03.2020–03.05.2020 (während der Pandemie)	Schwangere (*n* = 389) und Frauen in der postpartalen Phase (*n* = 186)	Prä-/postpartale Depression; peripartale Angst; PTBS	Großteil der Frauen: erhöhte Angst- und Depressionswerte während und nach der Schwangerschaft; schwangere Frauen mit Kindern wurden als vulnerabler eingestuft
				EPDS; WDEQ (A/B); PPQ	
Pariente et al. (2020), Israel	Kohortenstudie	18.03.2020–29.04.2020 (währende der Pandemie) November 2016 bis April 2017 (vor der Pandemie)	Frauen in der postpartalen Phase (*n* = 346)	Postpartale Depression	Geburten während der Pandemie: geringeres Risiko einer postpartalen Depression
				EPDS	
Perzow et al. (2021), USA	Längsschnitt	13.04.2020–22.05.2020 (während der Pandemie) Vor der Pandemie (Daten nicht angegeben) Während der frühen Schwangerschaft vor der Pandemie (Daten nicht angegeben)	Schwangere und Frauen in der postpartalen Phase (*n* = 135)	Prä-/postpartale Depression; Angst	Während Pandemie: höhere depressive und angstbezogene Symptome als zuvor; Depressionssymptome während der frühen Schwangerschaft (vor der Pandemie) waren genauso hoch wie während der Pandemie; Angstsymptome waren während der Pandemie höher als in der frühen Schwangerschaft (vor der Pandemie)
				EPDS; STAI-SF (Kurzform des STAI)	
Preis et al. (2020), USA	Querschnitt	25.04.2020–15.05.2020 (während der Pandemie)	Schwangere Frauen (*n* = 4451)	Stress	Erhöhte Werte: „preparedness stress“ und „perinatal infection stress“; Risikofaktoren für vermehrten Stress; Hochrisikoschwangerschaft, das Gefühl, infiziert worden zu sein und eine schwangere „person of color“ (PoC) zu sein
				PREPS	
Ravaldi et al. (2020), Italien	Querschnitt	18.03.2020–31.03.2020 (während der Pandemie)	Schwangere Frauen (*n* = 737)	COVID-19-bezogene Angst; PTBS	Positive Korrelation zwischen vergangenen Depressionen und Angststörungen mit PTBS-Symptomen; frühere Angststörungen sagten höhere COVID-19-bezogene Ängste voraus
				COVID-ASSESS; NSESSS	
Ravaldi et al. (2021), Italien	Querschnitt	März bis Mai 2020 (während der Pandemie)	Schwangere Frauen (*n* = 200)	COVID-19-bezogene Angst	Während der Pandemie: Angst die häufigste Emotion bezüglich der Geburt; sign. Korrelation früherer psychischer Störungen mit höheren COVID-19-bezogenen Ängsten
				COVID-ASSESS	
Wu et al. (2020), China	Querschnitt	20.01.2020–09.02.2020 (nach Ausrufung der Pandemie) 01.01.2020–20.01.2020 (vor Erklärung der Pandemie)	Schwangere Frauen während des 3. Trimesters (*n* = 4124)	Peripartale Depression; Angst	Während der Pandemie: sign. höhere Depressions- und Angstwerte; pos. Korrelation von COVID-19-Fallzahlen mit Depressionsraten
				EPDS; EPDS-3A	
Yang et al. (2021), China	Querschnitt	25.02.202–10.03.2020 (während der Pandemie)	Schwangere Frauen (*n* = 19.515)	Depression; generalisierte Angst	44,6 % bzw. 29,2 % der Frauen: klinisch auffällige Depressions- und Angstsymptome
				2 Items des soziodemografischen Fragebogens; PHQ‑9; GAD‑7	

#### Auswirkungen von COVID-2019 auf die Prävalenz und Schwere psychischer Erkrankungen

##### Stress.

Drei Untersuchungen machten Angaben zu Stress während der Schwangerschaft (Effati-Daryani et al. 2020; Matvienko-Sikar et al. 2020; Preis et al. 2020). Die Prävalenzen bewegten sich zwischen 27,2 % (Preis et al. 2020) und 32,7 % (Effati-Daryani et al. 2020). In einer Studie gaben 30 % der Teilnehmenden an, sowohl hohen Preparedness stress als auch hohen Perinatal infection stress erlebt zu haben (Preis et al. 2020). In der zweiten Studie wiesen schwangere Frauen während der COVID-19-Pandemie ein höheres Stressniveau auf als vor der Pandemie (Matvienko-Sikar et al. 2020). Dieser Anstieg war jedoch nicht signifikant. In einer iranischen Untersuchung gaben schwangere Frauen ähnliche Stresswerte während wie vor der COVID-19-Pandemie an (Effati-Daryani et al. 2020).

##### Angst und Depressionen.

Unterschiedliche Dimensionen von Angst während der Schwangerschaft und der Postpartalzeit erhoben16 Studien. Davon untersuchten 7 Studien die Facette generelle Angst (Basu et al. 2021; Berthelot et al. 2020; Cameron et al. 2020; Effati-Daryani et al. 2020; Lebel et al. 2020; McMillan et al. 2021; Yang et al. 2021). Alle Autoren berichteten von erhöhten Angstwerten während der COVID-19-Pandemie. Die Prävalenzraten variierten zwischen 29,2 % (Yang et al. 2021) und 56,6 % (Lebel et al. 2020). Drei Studien fanden signifikant höhere Angstsymptome bei schwangeren Frauen während der Pandemie im direkten Vergleich zu erhobenen Daten ähnlicher Kohorten vor dem Pandemieausbruch (Berthelot et al. 2020; Cameron et al. 2020; Lebel et al. 2020). Die spezifische COVID-19-bezogene Angst wurde in 3 Arbeiten erhoben (Dule et al. 2021; Ravaldi et al. 2020, 2021). Die Angst vor einer COVID-19-Infektion korrelierte in einer Studie moderat negativ mit der Lebensqualität der Frauen und sagte zudem eine signifikant geringere Lebensqualität voraus (Dule et al. 2021). Frühere Angststörungen waren in 2 italienischen Studien ein Prädiktor für ein stärkeres Ausmaß der COVID-19-bezogenen Ängste (Ravaldi et al. 2020, 2021). Sechs Studien erhoben prä- und peripartale Ängste (Cameron et al. 2020; Gonzalez-Garcia et al. 2021; Lebel et al. 2020; Liu et al. 2020; Molgora und Accordini 2020; Wu et al. 2020). Dort waren die geburtsspezifischen Angstwerte der Frauen deutlich erhöht. Bei mehr als ein Drittel der teilnehmenden Frauen überschritt den klinischen Cut-off-Wert der Perinatal Anxiety Screening Scale (PASS; Cameron et al. 2020). Außerdem gaben in einer weiteren Studie mehr als die Hälfte der schwangeren Frauen an, während der Pandemie klinisch signifikante Angst vor der Geburt zu erleben (Liu et al. 2020). In einer Studie korrelierte die Sorge über die Auswirkungen von COVID-19 auf das Baby signifikant mit Angst- und Depressionswerten (Gonzalez-Garcia et al. 2021). Zudem unterlagen erstgebärende Frauen einem höheren Risiko, Angstsymptome zu entwickeln als Frauen, die bereits ein oder mehrere Kinder hatten (Lebel et al. 2020; Wu et al. 2020). Im Gegensatz dazu wurden in einer Studie Frauen bezüglich der Angst vor der Geburt als vulnerabler eingestuft, wenn sie bereits Kinder hatten (Molgora und Accordini 2020). In der chinesischen Studie hatten schwangere Frauen in Wuhan (Epizentrum der Coronapandemie) signifikant höhere Angstwerte als in Chongqing (Gebiet mit geringerem Risiko; Liu et al. 2020).

In 13 Publikationen wurden Ergebnisse im Zusammenhang mit depressiven Symptomen berichtet, davon untersuchten 6 Studien Depression i. Allg. (Basu et al. 2021; Berthelot et al. 2020; Effati-Daryani et al. 2020; López-Morales et al. 2021; McMillan et al. 2021; Yang et al. 2021). Fünf der 6 Studien berichteten erhöhte Depressionswerte während der Pandemie. Die angegebenen Prävalenzraten schwankten zwischen 31 % (Basu et al. 2021) und 44,6 % (Yang et al. 2021). Die spezifische prä- und postpartale Depression wurde in 6 Studien erhoben (Cameron et al. 2020; Lebel et al. 2020; Molgora und Accordini 2020; Pariente et al. 2020; Perzow et al. 2021; Wu et al. 2020). Einige Autoren gaben separate Prävalenzen für schwangere Frauen (34,2 %) und für Frauen in der postpartalen Phase (26,3 %) an (Molgora und Accordini 2020). Fünf Studien verzeichneten eine Zunahme depressiver Symptome während der Coronapandemie (Cameron et al. 2020; Lebel et al. 2020; Molgora und Accordini 2020; Perzow et al. 2021; Wu et al. 2020). Demgegenüber berichtete eine Studie, dass Frauen, die während der Lockdownphase der Pandemie entbunden wurden, einem geringeren Risiko ausgesetzt waren, eine postpartale Depression zu entwickeln, als Frauen vor der Pandemie (Pariente et al. 2020).

##### Posttraumatische Belastungsstörung.

Symptome einer PTBS bei schwangeren und Frauen in der postpartalen Phase wurden in 5 Arbeiten untersucht (Basu et al. 2021; Berthelot et al. 2020; Gonzalez-Garcia et al. 2021; Molgora und Accordini 2020; Ravaldi et al. 2020). Die Prävalenzraten betrugen zwischen 10,2 und 43 %. Vier der 5 Studien fanden erhöhte PTBS-Werte während der Pandemie (Basu et al. 2021; Berthelot et al. 2020; Molgora und Accordini 2020; Ravaldi et al. 2020). In einer Studie war die PTBS-Prävalenz ähnlich hoch wie vor der Pandemie (3 %; Gonzalez-Garcia et al. 2021). Lediglich einige Symptome einer PTBS (negative Stimmung und Kognitionen) nahmen während der Pandemie zu (Berthelot et al. 2020). Spezifischere Symptome wie Intrusionen und Vermeidung traumabezogener Aspekte nahmen hingegen nicht zu.

#### Risiko- und Schutzfaktoren

Die Risiko- und Schutzfaktoren, die während der Pandemie zur Entstehung bzw. Vorbeugung einer postpartalen psychischen Erkrankung beitragen können, sind in Tab. 2 aufgeführt. Diese Übersicht spiegelt lediglich eine Momentaufnahme wider und ist keinesfalls allumfassend. Deutlich wird, dass finanzielle und intrafamiliäre Schwierigkeiten eher als allgemein sozioökonomische, gynäkologische oder gesundheitsbezogene Probleme Risiken für die Entwicklung psychischer Erkrankungen während der Pandemie darstellten. Die COVID-19-bezogenen Sorgen über das Kind und dessen Gesundheit sowie Sorgen hinsichtlich der medizinischen Versorgung während der Pandemie förderten Angsterkrankungen, depressive und Belastungssymptome. Als größte protektive Faktoren fungierten die Zufriedenheit mit dem Eheleben/eine höhere Qualität der Paarbeziehung und die physische Aktivität/der Zugang zu Außenbereichen. In Bezug auf prä- und postpartale Angsterkrankungen war die von den Frauen wahrgenommene soziale Unterstützung in 3 Studien ein sehr wirksamer Schutzfaktor. Explizite Faktoren zum Schutz vor der Entstehung einer PTBS wurden nicht genannt.

**Table 2:** 

Psychische Symptome	Risikofaktoren	Schutzfaktoren
Stress während der Schwangerschaft	Geringe physische Aktivität; Gewalt in der Partnerschaft (verbal und/oder physisch); Schwierigkeiten, Rechnungen zu bezahlen; direkte Betreuung von COVID-19-infizierten Personen; selbstangegebener Risikoschwangerschaftsstatus; chronische Erkrankung; früherer Missbrauch; COVID-19-bedingter Einkommensverlust; das Gefühl, infiziert worden zu sein, und Veränderungen in der Schwangerschaftsvorsorge; geringe wahrgenommene soziale Unterstützung (Matvienko-Sikar et al. 2020; McMillan et al. 2021; Preis et al. 2020) „Perinatal infection stress“: eine schwangere Woman of Color zu sein, das Fehlen einer Ehe oder Lebenspartnerschaft und früherer Schwangerschaftsverlust (Preis et al. 2020) „Preparedness stress“: erstgebärende Frauen, ungeplante Schwangerschaft und Mehrlingsschwangerschaft (Preis et al. 2020)	Zufriedenheit mit dem Eheleben, geringeres Bildungsniveau des Ehemannes, Verbleib in Isolation, höheres Alter und der Zugang zu Außenbereichen (Effati-Daryani et al. 2020; McMillan et al. 2021; Preis et al. 2020)
Angst	Finanzielle Belastung; familiäre Stressoren (im letzten Monat); Sorgen um das eigene Leben und das des ungeborenen Kindes; Sorgen um die Schwangerschaftsvorsorge; Symptome einer Infektion; vergangene psychische Erkrankungen (Cameron et al. 2020; Lebel et al. 2020; Liu et al. 2020; Molgora und Accordini 2020)	Höheres Maß an physischer Aktivität (Lebel et al. 2020); viel Wissen über COVID-19; eine rationale Risikowahrnehmung (Liu et al. 2020); Glaube an die Anwesenheit des Vaters bei der Geburt (Molgora und Accordini 2020); hohes Ausmaß wahrgenommener sozialer Unterstützung (Gonzalez-Garcia et al. 2021; Lebel et al. 2020; Yang et al. 2021)
Depression	Kürzlich belastende Erfahrungen (familiäre Stressoren); Sorgen um das eigene Leben und das des ungeborenen Kindes; Sorge, nicht die notwendige medizinische Versorgung zu erhalten; belastende Paarbeziehung; soziale Isolation und erhöhte Einsamkeit; mehr als ein Kind zu haben; vergangene psychische Erkrankungen; der Glaube, dass der Partner während der Geburt nicht anwesend sein würde (Cameron et al. 2020; Gonzalez-Garcia et al. 2021; Lebel et al. 2020; Molgora und Accordini 2020; Perzow et al. 2021)	Höhere Qualität der Paarbeziehung; physische Aktivität; Zufriedenheit mit dem Eheleben; geringeres Bildungsniveau des Ehemannes (Cameron et al. 2020; Lebel et al. 2020; Effati-Daryani et al. 2020)
PTBS	Übermäßige pandemiebezogene Informationssuche (5-mal oder öfter pro Tag); Sorgen in Bezug auf eigene Kinder und die medizinische Versorgung; ökonomische Sorgen; Sorgen in Bezug auf eine COVID-19-Infektion; Sorgen über die Entbindung; räumliche Distanzierung zum Infektionsschutz; psychiatrische Vorgeschichte; geringeres Haushaltseinkommen; vergangene psychische Erkrankung; Partner/in bei der Geburt nicht anwesend; Komplikationen während der Geburt (Basu et al. 2021; Berthelot et al. 2020; Molgora und Accordini 2020; Ravaldi et al. 2020)	–

## Diskussion

### Interpretation der Ergebnisse

Allgemein ist bekannt, dass Epi- und Pandemien sowohl die Bevölkerung als auch die Gesundheitssysteme vor starke Herausforderungen stellen (Brooks et al. 2020). Ziel dieser Übersichtsarbeit war es, den aktuellen Forschungsstand zu den Auswirkungen der COVID-19-Pandemie auf die psychische Gesundheit von schwangeren Frauen und von Frauen nach der Geburt zusammenzufassen. Zudem sollten potenzielle Risiko- und Schutzfaktoren identifiziert werden, die zur Entstehung bzw. zur Vorbeugung von postpartalen psychischen Erkrankungen beitragen. Insgesamt wurden 19 Publikationen mit 44.709 Teilnehmerinnen berücksichtigt.

Ein negativer Einfluss der pandemiebezogenen Belastungen wurde sichtbar. Beinahe alle Studien verzeichneten einen deutlichen Anstieg der psychischen Symptome. Vor allem Ängste vor einer COVID-19-Infektion korrelierten moderat negativ mit der Lebensqualität der Frauen (Dule et al. 2021). Hinsichtlich der postpartalen Ängste überschritt ein Drittel der Frauen den klinischen Cut-off-Wert der PASS (Cameron et al. 2020). Auch im Bereich der prä- und postpartalen Depressionswerte wurde überwiegend ein Anstieg verzeichnet (u. a. Basu et al. 2021; Berthelot et al. 2020; Effati-Daryani et al. 2020; López-Morales et al. 2021; McMillan et al. 2021; Yang et al. 2021). Die PTBS-Werte während der Pandemie stiegen in 4 Studien signifikant (Basu et al. 2021; Berthelot et al. 2020; Molgora und Accordini 2020; Ravaldi et al. 2020).

Insgesamt weisen die Ergebnisse darauf hin, dass schwangere Frauen sowie Frauen in der Postpartalzeit während Pandemien einer besonderen Belastung unterlagen. Depressionswerte, Ängste und PTBS-Symptomatik scheinen im Zusammenhang mit COVID-19 im Vergleich zu früher berichteten Bevölkerungsnormen erhöht zu sein. Dies ist konsistent mit den bisherigen Forschungen (Campos-Garzón et al. 2021; Cigăran et al. 2021). In einem Scoping-Review zu den psychologischen Auswirkungen der COVID-19-Pandemie auf schwangere Frauen zeigten 40 % der Befragten posttraumatische Belastungssymptome. Wiederum 70 % der Frauen litten unter Depressionen und/oder Angsterkrankungen (Campos-Garzón et al. 2021).

In Bezug auf den empfundenen Stress wurden in der vorliegenden Arbeit weniger starke Auswirkungen von COVID-19 angegeben als erwartet. Lediglich in 3 der 19 Studien wurden Angaben zu Stress während der Schwangerschaft gefunden (Effati-Daryani et al. 2020; Matvienko-Sikar et al. 2020; Preis et al. 2020). In einer amerikanischen Studie gaben die Teilnehmenden an, sowohl hohen Preparedness stress als auch hohen Perinatal infection stress erlebt zu haben (Preis et al. 2020). In der zweiten Studie wiesen schwangere Frauen während der Pandemie ein höheres Stressniveau auf (Matvienko-Sikar et al. 2020). Dieser Anstieg war jedoch nicht signifikant. Zuletzt ergab eine Untersuchung ähnliche Stresswerte wie vor der COVID-19-Pandemie (Effati-Daryani et al. 2020). Eine mögliche Erklärung könnte sein, dass Schwangerschaften generell als ein „life event“ gesehen werden und daher mit einem erhöhten Belastungserleben und erforderlicher Adaptationsleistung assoziiert ist (Geller 2004). Weiterhin muss berücksichtigt werden, dass wahrscheinlich nicht die gesamte Bandbreite aktueller Studien, die Stress bei schwangeren Frauen und postpartalen Müttern während der Pandemie erhoben haben, aufgenommen wurde.

Als bedeutsamste Risikofaktoren wurden v. a. finanzielle (u. a. Einkommensverluste, geringes Haushaltseinkommen) und intrafamiliäre Einflüsse (Gewalt in der Partnerschaft, belastende Paarbeziehungen) identifiziert. Aber auch gesundheitliche Faktoren (frühere psychische Erkrankungen, chronische Erkrankungen) und „medizinische“ Faktoren, die das Gesundheitssystem betreffen (Veränderungen in der Schwangerschaftsvorsorge, das Fehlen der notwendigen medizinischen Versorgung), spielen bei dem Erleben psychischer Belastung eine Rolle (Tab. 2). Diese Ergebnisse stimmen mit bisherigen Ergebnissen überein (Cigăran et al. 2021). Vor allem im medizinischen Bereich nahmen die Sorgen der schwangeren Frauen über die eigene Gesundheit, über die des ungeborenen Kindes sowie die Angst vor einer Ansteckung mit COVID-19 während der Pandemie zu (Naurin et al. 2021). Psychische Erkrankungen und erhöhter Stress der Mutter während der Schwangerschaft können negative Folgen für die Gesundheit des Babys haben (u. a. Kramer et al. 2009; Van den Bergh et al. 2020; Zietlow et al. 2019). Ein zu hoher Kortisolspiegel der Mutter führt zu Einschränkungen in der kindlichen Entwicklung (Rafael et al. 2022). Dazu gehören ein niedriges Geburtsgewicht, eine verringerte neuronale Entwicklung oder das Auftreten einer pathogenen Darmflora. In einer aktuellen Studie zeigten Mütter in der COVID-19-Gruppe stark erhöhte Depressionswerte, die nach der Geburt in Verbindung mit Regulationsschwierigkeiten des Babys standen (Perez et al. 2022). Auch das Ausschließen des Partners bei der Geburt während der Pandemie stellte einen hohen Risikofaktor da (Naurin et al. 2021).

Als einflussreicher, aber zu wenig untersuchter Schutzfaktor, stellte sich in dieser Arbeit die wahrgenommene soziale Unterstützung heraus. Soziale Unterstützung spielt in vielen sozialpsychologischen und gesellschaftlichen Kontexten eine wichtige Rolle. Interpersonelle Unterstützung und Hilfe von FreundInnen, Familie oder PartnerInnen gilt als fundamentaler Schutzfaktor für die physische und psychische Gesundheit. Zudem hat sie eine positive Wirkung auf die Resilienz einer Person (Dorsch et al. 2017) Die wahrgenommene soziale Unterstützung schwangerer Frauen bietet Schutz gegen postpartale psychische Erkrankungen und mindert gleichzeitig negative Aspekte der Geburtserfahrung (Tani und Castagna 2017). In 3 Studien war ein höheres Level an wahrgenommener sozialer Unterstützung ein Schutzfaktor für depressive Symptome und Angst (Gonzalez-Garcia et al. 2021; Lebel et al. 2020; Yang et al. 2021). Darüber hinaus korrelierte wahrgenommene soziale Unterstützung signifikant negativ mit den COVID-19-bezogenen Sorgen über das Neugeborene und allgemeinen Sorgen bezüglich COVID-19 (Gonzalez-Garcia et al. 2021). Zukünftige Untersuchungen sollten den Einfluss der wahrgenommenen sozialen Unterstützung während der Schwangerschaft noch näher betrachten.

Die Ergebnisse zeigen deutlich, dass schwangere Frauen und Frauen in der Postpartalzeit stärker im Fokus des Gesundheitssystems stehen sollten. Hierzu gehören einerseits der Ausbau von ambulanten und stationären Nachsorge‑, Beratungs- und Therapieangeboten für betroffene Frauen sowie ihre Kinder (Hübner-Liebermann et al. 2012). Andererseits sollten im Hinblick auf das erhöhte Risiko von Partnerschaftsgewalt während der COVID-19-Pandemie Routineuntersuchungen auf physischen und emotionalen Missbrauch eine entscheidende Rolle spielen (Froimson et al. 2020). Weiterhin sollten geeignete Präventions- und Interventionsmöglicheiten entwickelt werden, um Frauen mit postpartalen psychischen Erkrankungen zu helfen. Hierbei sollten nicht nur die betroffenen Frauen die Zielgruppe darstellen, sondern ebenso das medizinische Personal und Ärzte in den Kreissälen, Hebammen sowie PartnerInnen und Familien.

### Stärken und Limitationen der Studie

Die Ergebnisse des vorliegenden Reviews müssen unter Einbezug der Stärken und Limitationen interpretiert werden. Zunächst ist positiv hervorzuheben, dass die Arbeit auf einer systematischen Literatursuche basiert und einen ersten Beitrag zum Forschungsstand in Deutschland liefert. Einschränkend muss erwähnt werden, dass keine Qualitätsbewertung der Einzelstudien durchgeführt wurde und die Arbeit daher einen narrativen Charakter erhält. Die wenig restriktiven Ein- und Ausschlusskriterien führten zu einer enormen Heterogenität der ausgewählten Studien, sodass ein Vergleich der Ergebnisse nur eingeschränkt möglich ist. Ein überwiegend querschnittliches Design der eingeschlossenen Studien sowie unterschiedliche Erhebungszeiträume zu Beginn der COVID-19-Pandemie schränken die Vergleichbarkeit weiter ein. Des Weiteren bestanden die Online-Umfragen beinahe ausschließlich aus retrospektiven Selbsteinschätzungen, die durch verschiedene Einflüsse verzerrt sein können. Wie in diesem Review festgestellt werden konnte, scheint die wahrgenommene soziale Unterstützung ein protektiver Faktor hinsichtlich der Entwicklung von peri-, aber auch postpartalen Angsterkrankungen zu sein. Da das Konstrukt der wahrgenommenen sozialen Unterstützung selbst nicht als Variable aufgenommen wurde, wäre dies für weitere Untersuchungen wünschenswert. Abschließend sollte darauf hingewiesen werden, dass ein breiteres gesellschaftliches Spektrum an Diversität wünschenswert ist, auch, um eine mögliche Konfundierung mit dem Konstrukt Minderheitenstress zu vermeiden.

## Fazit für die Praxis

- Symptome von Angst, Depressionen und Belastungsstörungen nahmen während der COVID-19-Pandemie überwiegend zu.
- Eher finanzielle, familiäre und medizinische als allgemein sozioökonomische und gynäkologische Risikofaktoren waren für die Entwicklung prä- und postpartaler psychischer Erkrankungen während der Pandemie mitverantwortlich. Als protektive Faktoren fungierten die Zufriedenheit mit der Paarbeziehung und physische Aktivität/Zugang zu Außenbereichen.
- Wahrgenommene soziale Unterstützung als Schutzfaktor bei prä-/postpartalen Erkrankungen sollte näher untersucht werden.
- Trotz fortlaufender Forschung gibt es bisher kaum Beratungs- oder Therapieangebote mit Fokus auf die Postpartalzeit.
- Nachsorge- und Screeningverfahren sind in der Postpartalzeit besonders relevant für die Prävention schwerer Erkrankungsverläufe.
- Ergänzend wird empfohlen, Kinder, die während der Pandemie geboren und aufgewachsen sind, hinsichtlich psychischer Auffälligkeiten zu beobachten und spezielle Therapieangebote zu stellen.
