# Erratum zu: Einfluss der COVID-19-Pandemie auf die psychische Gesundheit während der Peripartalzeit – eine narrative Übersicht

**DOI:** 10.1007/s00278-023-00664-8

**Published:** 2023-06-05

**Authors:** S. Gries, N. S. Teichmann, F. M. L. Beck-Hiestermann, B. Strauß, A. Gumz

**Affiliations:** 1 Psychologische Hochschule Berlin, Am Köllnischen Park 2, 10179 Berlin, Deutschland; 2 Institut für Psychosoziale Medizin, Psychotherapie und Psychoonkologie (IPMPP), Universitätsklinikum Jena, Jena, Deutschland

**Erratum zu:**

**Psychotherapie 2023**

10.1007/s00278-023-00646-w

In diesem Artikel fehlte N. S. Teichmann an der Psychologische Hochschule Berlin, Berlin, Deutschland in der Autorenliste. Die korrekte Autorenliste lautet: S. Gries, N. S. Teichmann, F. M. L. Beck-Hiestermann, B. Strauß, A. Gumz. Der Interessenkonflikt wurde ebenfalls ergänzt: N. S. Teichmann gibt an, dass kein Interessenkonflikt besteht.

In diesem Artikel waren außerdem die Adressdaten für den Autor B. Strauß falsch angegeben. Statt ‚Friedrich-Schiller-Universität Jena, Jena, Deutschland‘ hätte sie ‚Universitätsklinikum Jena, Institut für Psychosoziale Medizin, Psychotherapie und Psychoonkologie (IPMPP), Jena, Deutschland‘ lauten sollen.

Der Originalbeitrag wurde korrigiert.

